# A Conceptual Framework Toward Understanding of Knowledge Acquisition Sources and Student Well-Being

**DOI:** 10.3389/fpsyg.2020.01852

**Published:** 2020-09-10

**Authors:** Yan Xu, Michael Yao-Ping Peng, Yangyan Shi, Shwu-Huey Wong, Wei-Loong Chong, Ching-Chang Lee

**Affiliations:** ^1^Business College, Yango University, Fuzhou, China; ^2^College of Management, National Kaohsiung University of Science and Technology, Kaohsiung, Taiwan; ^3^School of Economics & Management, Foshan University, Foshan, China; ^4^School of Digital Economics, Guilin University of Electronic Technology, Guilin, China; ^5^Business School, Guilin University of Technology, Guilin, China; ^6^Department of Education, New Era University College, Kajang, Malaysia; ^7^Department of Information Management, National Kaohsiung University of Science and Technology, Kaohsiung, Taiwan

**Keywords:** teacher knowledge transfer, market orientation, student employability, absorptive capacity, self-efficacy, well-being

## Abstract

There are a multitude of factors influencing student employability, with all previous studies basing their conclusions upon predetermined variables according to different theories and exploring the relevance between them. In this study, teachers’ knowledge transfer and market orientation—are put forward on the basis of the marketing concepts in order to explore the conspicuousness between various factors within the structural model. This study uses students from colleges in Taiwan and mainland China, and purposive sampling is adopted to acquire samples required for statistics. A total of 1,222 valid questionnaires were collected from Taiwanese and Mainland China students. The results indicate that knowledge transfer, market orientation and absorptive capacity have significant impacts on student employability, that the absorptive capacity has a positive moderating effect on the influence of knowledge transfer and market orientation on student employability. Based on results and findings, this study will provide suggestions for theoretical and practical implications.

## Introduction

In this era of the knowledge economy, knowledge and capability-based views assert that knowledge is the main source of enterprise innovation, new value creation, differentiation and access to competitive advantage (Zhou and Li, 2012). A focus on higher education is likewise relevant; the importance of knowledge to college students is at the core of personal differentiation and personal value creation. How to use knowledge to enhance their competitiveness to enter the job market is an important issue to be discussed (Chung et al., 2016). In the field of higher education, most studies explore factors that affect student’s learning effectiveness (Pike et al., 2011, 2012) or the application effects of learning patterns (Pascarella et al., 2013). However, few studies have explored the knowledge-processing of students from the “input-process-output” stance of knowledge management with regards to the future entry of students into the workplace (Baek and Cho, 2018). The ability to utilize and apply internalized knowledge is to cultivate a student’s employability (Blázquez et al., 2018). Some scholars argue that “employability” is an individual’s need to continuously acquire or create jobs through appropriate use of competence, to accomplish tasks and adapt to changes in the internal and external labor market (Fugate et al., 2004; Cuyper et al., 2008; Vermeulen et al., 2018; Shahzad et al., 2020). According to the cognitive learning theory, the development of employability can be regarded as the process of forming knowledge cognition, and the acquisition and input of knowledge is the source of learning stimulation.

A majority of studies on higher education have discussed factors influencing learning outcomes of students (Pike et al., 2012; Bailey and Phillips, 2016) or the application effect of learning models (Pascarella et al., 2013; Campbell and Cabrera, 2014). Some studies in recent years began to discuss the shape of student well-being from the view of educational psychology. The emergence of positive psychology leads the psychology (Seligman and Csikszentmihalyi, 2000) into a new direction. Under the influence of the positive psychology, counseling and psychotherapy begin to turn their attention to positive affect subject (Stallman et al., 2018). Many scholars advocate to emphasize the well-being of adolescent, and believe that well-being is the core of adolescent’s mentally healthy development (Miller et al., 2008; Graham et al., 2016). This study replaces student learning outcome with student well-being as the core view: (1) Well-being, as the major concerns of student personality and social psychology, is used to examine social change and improvement of educational policies and solve student learning problems (Bailey and Phillips, 2016; Hanson et al., 2016; Stallman et al., 2018). (2) The discussion of student well-being will put emphasis on finding symptoms such as possible depression, anxiety, and psychological disorder (Bailey and Phillips, 2016) the positive and negative psychology lies between two extremes of continuous psychological states, and the more well-being of students will help students face challenges with a positive psychological state, and increase the value of learning course (Stallman et al., 2018). Considering the above reasons, this study aims to further understand and discuss the development course of student well-being through enhancing students’ employability in the learning process.

From a system perspective, the source of knowledge acquisition can be divided into internal and external. Webster and Hommond (2008) point out that the development of university education standards should be conjoined with the market orientation concept of marketing theory (Narver and Slater, 1990; Webster and Hommond, 2008; Webster et al., 2014). Universities need to continuously perceive the needs of students—both at present and in the future—to help them understand the advantages and conditions of off-campus competitors, and to create, communicate and balance the values of students and related stakeholders (Webster et al., 2014). According to the above phenomena, the purpose of this study is to explore the impact of teacher knowledge transfer (TKT) and market orientation (MO) on student employability (SE).

Although students can attain valuable knowledge and information from teachers and external markets, they are not sure to develop high-quality employability. While market-oriented research suggests that systematic acquisition and analysis of information and knowledge contributes to capacity and performance (Slater and Narver, 1995) this argument seems to ignore exactly how students use this valuable knowledge. Fabrizio (2009) emphasizes connectedness—students should have some level of method and mechanism to learn, digest, transfer and apply knowledge, and enhance the benefits of that knowledge; namely, absorptive capacity (AC) (Cohen and Levinthal, 1990). Despite the abundance of rich and valuable knowledge and information, the AC of students is of crucial importance. Therefore, this study uses AC as the moderating variable between knowledge acquisition and employability, and understands its impact on employability.

Scholars believe that the results of a cross-country comparison can provide insights with profound implications; as such, scholars often use cross-cultural methods to compare research differences in different contexts (Chang et al., 2011). There are differences in the development of student well-being in the context of higher education of different countries, even if in Asian regions (Jang et al., 2016; Shogren et al., 2018; Al-Jubari et al., 2019). Although mainland China and Taiwan are difference in higher education policies, cultural distance and language teaching also show similarities. That’ why students of mainland China and Taiwan are used as samples in this study for comparative analysis. According to the above explanations, this study intends to propose relevant research contributions based on the following theoretical gaps: (1) applying learning cognition theory to explore students’ knowledge acquisition in higher education; (2) exploring the capability/skill development from the perspective of students to cultivate and establish students’ employability, and verify the relevance among sources of knowledge acquisition, absorptive capacity and employability; (3) adding the cross-country comparisons to research framework to explore the difference in subjective well-being of students in mainland China and Taiwan.

## Literature Review and Hypotheses Development

### Student Employability (SE)

The substantial technological, social, and economic changes that have occurred in recent decades have modified the concepts and operations of industrial organizations (Abbas and Saǧsan, 2019) and HEIs across the world (Vermeulen et al., 2018). Hence, dynamic HEIs maintain the highest standards of human capital development, so as to contribute to economic growth (Ahmed et al., 2015; Baek and Cho, 2018). Student employability drives the reform of higher education policies in various countries and has become the core project of university administration (Cranmer, 2006). The research of employability has been paid more and more attention by scholars, through research context and research method design in conjunction with cross-theory and practice analysis to understand the meaning of employability and its causal relationship with other factors (Hennemann and Liefner, 2010; Avramenko, 2012; Baek and Cho, 2018). There are a number of important factors that cannot be learned from higher education courses, such as personal conditions, interpersonal relationships, and external reasons (McQuaid and Lindsay, 2005).

It can be seen that employability is a social and psychological construct, including subjective and objective aspects (De Vos et al., 2011; Blázquez et al., 2018). In the objective aspect, The Department of Education, Science and Training (DEST) (2006) established an “employability skills framework” with eight categories: communication skills, teamwork ability, problem-solving ability, original and entrepreneurial ability, planning and organizational ability, self-management ability, autonomous learning, and scientific and technological ability. In the subjective aspect, some scholars have developed measurement scales to examine individual cognition on employability in several way (Andrews and Higson, 2008; Pan and Lee, 2011). The factors of employability need to consider elements such as national culture, industrial development and population structure (Lurie and Garrett, 2017; Likisa, 2018) and subjective aspect should be taken into account in this study. Pan and Lee (2011) based on the employability measurement scale developed by Andrews and Higson (2008) surveyed the flow of Taiwanese graduates, and found that employability can cover general ability for work, professional ability for work, attitude at work, career planning and confidence.

### Student Employability and Self-Efficacy

Social cognition scholars argue that individuals’ behavioral outcomes will be influenced by both environmental and cognitive factors in a given situation, especially those beliefs that lead to success and behavior (Lent et al., 2014; Wang et al., 2016). They call these beliefs “self-efficacy,” an important cognitive variable in personal factors during the process of interpreting individual formative behaviors, and interaction with the environment (Lent et al., 2014; Sheu et al., 2014). It can also be seen as the basis for human behavioral motivation, mental health and personal achievement (Dacre and Qualter, 2013). Self-efficacy is widely used in the field of education to explore the psychological cognitive factors of students of different ages and their positive impact on academic achievement and student career development (Wang et al., 2016).

According to the above discussion, students who have confidence in their abilities will have more efficient behavior and better interpersonal relationships than those who do not. According to Dacre and Qualter (2013) highly self-motivated students look for resources and opportunities to accomplish tasks that exist in social networks. Only by establishing and maintaining network relationships can they achieve their goals. Knowledge and resources are needed (Lent et al., 2014; Sheu et al., 2014). Furthermore, teamwork can also be seen as a strong network relationship, and the process of students solving problems and achieving tasks through teamwork will positively affect their employability. It is pointed out that, according to the above, this study proposes the following assumptions:

H1: Self-efficacy has a positive and significant impact on SE.

### SE and Subjective Well-Being (SWB)

People will eventually begin to reflect on the self-seeking of material satisfaction, further seeking psychological satisfaction and beginning to emphasize the importance of quality of life; thus the proposal of the concept of SWB. SWB is a result of satisfaction of life coupled with perceived positive and negative emotional intensity (Pearce, 2017). Keyes and Waterman (2003) and Keyes (2005) expanded the definition to incorporate the concept of “social well-being” by merging the two (psychological well-being and emotional well-being) to delineate SWB as a sum of three aspects: in the sense of psychological well-being, it serves to explore self-psychological adjustment and the macro-consciousness of the individual’s inner self; a sense of evaluating the function of the self in life through public and social norms; and lastly, emotional well-being as the individual’s awareness and assessment of the emotional state of self-life (Pearce, 2017).

Cuyper et al. (2008) considers employability as having its importance in the post-industrial knowledge society by continuously updating knowledge to maintain competitiveness in a global market (Griffeth et al., 2005) and making them feel capable of dealing with temporary and future developments—new psychological contracts created by individuals will likely increase their well-being. In addition, individuals can process the same things and tasks more efficiently and in less time with relevant experience, updated skills and knowledge—as well as a well-developed social network—so as to improve employability (Griffeth et al., 2005). The abundance of time saved will be used for life needs and personal future planning, thereby enhancing happiness. Similarly, students with higher employability can face the challenges of the future with a broader perspective. In addition to mastering the content of school work, they also have a more precise direction for planning and preparation for entering the workplace, reducing their insecurity and enhancing SWB. Based on the above phenomena, the hypothesis of this study is as follows:

H2: SE has a positive and significant impact on SWB.

### Self-Efficacy and Subjective Well-Being

Some scholars have focused their investigations on mental health concerns, social support, and coping styles in low SES college students (Tong and Song, 2004). However, few studies thus far have tapped this population’s general self-efficacy and SWB (Pearce, 2017). Tong and Song (2004) research findings indicated that low-SES college students reported a lower level of social support, limited sources of support, and low perceived support. It implies that low-SES college students’ general self-efficacy and SWB decrease because they are unable to receive timely and necessary psychological support when confronting stress. In addition, it might contribute to unique stressors. Conversely, students with higher self-efficacy have higher SWB (Pearce, 2017). In summary, the study infers the following:

H3: Self-efficacy has a positive and significant impact on SWB.

### Market Orientation (MO) and Student Employability

In particular, Narver and Slater (1990) define “market-oriented” as a composition culture that creates value for customers efficiently and effectively, thereby establishing superior performance for the company. They propose three cultural measurement aspects: (1) “customer orientation,” which means that students can understand the requirements and expectations of future employers from the perspective of employment; (2) “competitor orientation,” an analysis of the existing and potential graduates of other universities, to understand their short-term advantages and disadvantages as well as long-term possible development capabilities and strategies; and (3) “inter-functional coordination,” meaning that the university can provide the target employer with the value of future superior employees (i.e., graduates) through the integration and application of on-campus curriculum and administrative resources. Even the Education Criteria for Performance Excellence of the Malcolm Baldrige National Quality Award (MBNQA) raises the importance of MO for the development of higher education (Webster and Hommond, 2008; Webster et al., 2014).

Regarding employability, Fugate et al. (2004) found that it is a multidimensional construct covering career identity, personal adaptability, social and human capital etc., which helps to create individual employability; that is, from a learning perspective, it emphasizes individual initiative learning and engaging in activities or participating behaviors to promote self-adjustment and enhance the possibility of strategic change and success (Crossman and Clarke, 2010). The study pointed out that in the process of constructing individual employability, collecting employment information is a necessary skill; students should be aware of the employment situation in the labor market, including familiarity with industrial structure and workplace information so as to understand the ways to effectively obtain work and create social capital to obtain job opportunities and important resource conditions (Bridgstock, 2009). The establishment and development of relevant beliefs, values and cultures in this kind of market-sensing orientation will encourage students to actively collect employment information, explore careers, select suitable industries, and propose an energy-increasing plan to build individual employment competitiveness.

H4: Market-oriented culture has a positive and significant impact on SE.

### Teacher Knowledge Transfer (TKT) and Student Employability

According to the cognitive learning perspective, students can use their abilities and resources to build their own core competencies and shape their employability through the use of their abilities. Whether it is the acquisition, accumulation or creation of knowledge, each will certainly be affected by the process of knowledge transfer; only knowledge transfer can effectively create new knowledge to be applied in the management process to create value for individuals (Walter et al., 2007). Therefore, in the learning processes of knowledge recipients, the information must be internalized. Knowledge exists in the human mind through learning or experience, and then gradually grows with experience, involving personal beliefs, judgments, and value perceptions, in addition to explicit textual behavior, including the implicit mental journey. Polanyi (1962) distinguishes between tacit knowledge and explicit knowledge. There are some differences between the two types of knowledge. Explicit knowledge is objective and rational, and it can be encoded and stored in various physical and electronic formats. Tacit knowledge is the individual’s own experience, reflection, cognition, or talents, which are difficult to be presented (Astorga-Vargas et al., 2017). Scholars suggested four steps of process of knowledge transfer: (1) socialization: the beginning of knowledge transfer with the process of tacit knowledge, facilitation of life experience, and the capacity among students where they reside and are needed. It happens regularly through meeting records, that modeling the way of work by repeating a task that leads others to learn by example; (2) externalization: this propitiates all activities that are grouped and aimed at capturing, organizing, structuring, representing, coding knowledge, in order to facilitate the management by changing the knowledge from tacit one to explicit one (Inkpen and Dinur, 1998; Nonaka and Von Krogh, 2009; Zhou et al., 2010) (3) combination: there is a process in which different pieces of existing explicit knowledge are merged to create a new explicit knowledge; (4) internalization: process is carried out by putting into practice what has been learned from explicit knowledge (Astorga-Vargas et al., 2017). According to the above description, this study defines TKT into a learning process. Teachers will pass implicit knowledge and explicit knowledge to students through knowledge externalization, enabling students to integrate it with their own currently held knowledge.

Teacher knowledge transfer helps students to learn richer knowledge. In addition to the teaching experience of teachers, it also needs the cooperation of the learning environment. As mentioned above, TKT is a learning process which includes changes in the learning environment, assignment of course tasks, or conversion of the teacher’s instruction style. According to the characteristics of TKT, although implicit knowledge is more difficult to transfer than explicit knowledge, teachers use learning patterns to assist students in acquiring the value of knowledge. In short, the use of explicit knowledge helps to enhance general work ability and professional work ability, while promoting the improvement of learning efficiency and effectiveness, thereby enhancing students’ work attitude and self-confidence. Explicit knowledge plays an indispensable role in general and professional competence, but the most important aspect of this its combination with implicit knowledge to enhance the energy of innovation. In addition, Teigland and Wasko (2009) believe that explicit TKT will help students to reuse knowledge, besides solving common problems, interaction with teachers, and creating new knowledge; the transfer and integration of implicit knowledge can also generate new ideas and novel solutions. Therefore, this study infers the following assumptions:

H5: TKT has a positive and significant impact on SE.

### Student Absorptive Capacity (SAC) as a Moderator

Nieto and Quevedo (2005) defined AC as the ability of companies to identify new values, acquire external knowledge, and absorb and apply this knowledge to commercial purposes. Based on existing knowledge, this AC serves to develop and promote new knowledge. In other words, in the context of higher education, the maintenance of students’ own abilities will determine how to apply, integrate, and even fundamentally develop their core competencies. Cadiz et al. (2009) pointed out that personal AC is the process of applying new knowledge through assessment (identification and filtering of valuable information), digestion (translating new knowledge into usable knowledge), and application (using knowledge and converting it into usable knowledge). When students have strong AC, this capacity will enable students to generate new ideas in the process of learning, and even enhance the efficiency of TKT in the process of teamwork to complete those tasks explained by teachers (Wang et al., 2016).

The composition of employability includes knowledge, skills, and abilities (Hennemann and Liefner, 2010). In the context of higher education, if there is no ability to absorb knowledge on the receiving end of the knowledge, even if the knowledge transmitted by the teacher or school avenue is of value, students may not be able to use this knowledge effectively. Students with sufficient AC will openly communicate and exchange knowledge content through common interests and language, and retain valuable knowledge (Cadiz et al., 2009). Its employability has a positive impact. Nor et al. (2012) pointed out that classifying students as having AC means that they have superior advanced knowledge and academic achievement, can effectively transfer knowledge and apply it, and improve their future academic achievement, thus enhancing their employability. Based on the above description, this study infers the following assumptions:

H6: Students’ AC has a positive and significant impact on SE.

Employability is composed of knowledge, technology, and diversity (Hennemann and Liefner, 2010; Baek and Cho, 2018). In the context of higher education, students may not have the knowledge and ability to absorb knowledge (Blázquez et al., 2018) even if the information provided by teachers is enriched. The market-oriented culture department emphasizes that students obtain relevant information from competitors and employers from the external employment market and respond to the information through the integration process. Therefore, the market-oriented culture is the mechanism for students to deal with external information. However, even if students are willing to collect external knowledge and information, SE is difficult to be improved if students have no sufficient basic knowledge and capabilities to absorb these knowledge and information, whereas AC plays an important role in the process of knowledge processing. Zahra and George (2002) argue that AC includes interpretation, comprehension, and learning. From the point of view of external knowledge, Szulanski (1996) argues that individuals’ external knowledge is affected by the environment in which they live (Shahzad et al., 2020). Accordingly, external knowledge will differ in its meaning and value when used in different environments and thus enhance one’s understanding, digestion, and replication of the knowledge (Blázquez et al., 2018; Abbas and Saǧsan, 2019). In other words, students with sufficient AC will be able to communicate and share knowledge about the connotation of knowledge through mutual interest and language, and then acquire valuable knowledge (Diener et al., 1999; Cadiz et al., 2009; Abbas and Saǧsan, 2019). Furthermore, in addition to facilitating students to receive and respond to future job market related information, competitors’ message processing and institutional administration can also help students to improve their learning planning and effectiveness (Webster and Hommond, 2008; Webster et al., 2014). Consequently, if students have adequate AC, a higher degree market-oriented culture will more effectively facilitate students to take into account the important changes in the job market, and their employability that they have cultivated can meet the needs of employers. Thus, a hypothesis is made as follows:

H7: Students’ AC has a positive effect on the relationship between market-oriented culture and SE.

Nieto and Quevedo (2005) argued that AC means the individual’s ability to acquire, digest, and apply knowledge because of its path-dependent nature; therefore, based on the knowledge stock accumulated by students in the past, the key traits are students’ ability to communicate with teachers or peers in a common language, share their unique knowledge, and apply new knowledge with each other. In the process of transforming external new knowledge, Yeoh (2009) believed that students will become embedded in the relationship of mutual cooperation and trust through the process of socialization. When the interaction between students and teachers increases, it will be helpful to improve the efficiency of communication and knowledge exchange between teachers and students. Jiang et al. (2008) believed that if students have better AC, they can improve their ability to use it, digest new knowledge more efficiently, and have a deeper understanding of new knowledge for enhancing their professional and general abilities. In other words, if students do not have such AC, they cannot completely digest and apply implicit or explicit knowledge transferred from the teacher’s side (Chen, 2003). Superior AC can promote students’ spontaneous learning behavior, identify and utilize their own abilities and resources, and integrate new external knowledge in order to promote their employability. Therefore, this study infers the following assumptions:

H8: Students’ AC has a positive effect on the relationship between TKT and SE

**FIGURE 1:**
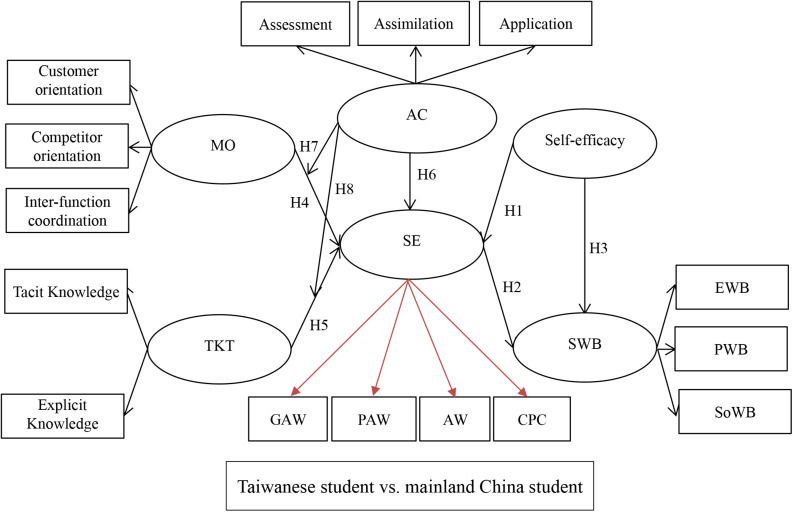
Research framework.

Based on the above hypothesis, this study proposes the following research framework as Figure 1 shown:

## Methodology

### Participants and Procedure

This study proposed a framework to explore the correlations and development mode of MO, TKT, AC, SE, self-efficacy and SWB. It sampled from Taiwanese and mainland China HEIs, including both public and private. This study also incorporates students’ year of study as a sampling condition, as freshmen were left out of sample. This study selected 12 Taiwanese HEIs and 6 mainland China HEIs, and then sent 2,000 questionnaires to each of them. After sampling, a total of 657 Taiwanese questionnaires and 565 mainland China questionnaires were returned, for an effective response rate of 65.7 and 54.8%. Since freshmen were not familiar with the learning environment, all participants in this study were sophomores, junior, and senior students. In this study, according to analysis method of Lin et al. (2014) we used two method tools to analyze research framework. First, we adopted SEM to test the direct path among variables. Second, we used hierarchical regression analysis to examine the moderating effect of AC with interaction items and several control variables.

### Measures

All constructs were measured by multiple-item scales based on previous studies. Similar to the employability scale reported by Pan and Lee (2011) eighteen items were used to capture general ability for work (GAW) (8 items), professional ability for work (PAW) (4 items), attitude at work (AW) (3 items) and career planning and confidence (CPC) (3 items). MO was adapted from Narver and Slater (1990) and Hammond et al. (2006) which was measured in terms of customer orientation (6 items), competitor orientation (4 items), and inter-functional coordination (6 items). TKT uses the explicit knowledge (5 items) and tacit knowledge (4 items) scales developed by Zhou et al. (2010). Following Cadiz et al. (2009) absorptive capacity was measured in terms of assessment (3 items), assimilation (3 items) and application (3 items). In self-efficacy, six items were selected on the basis of prior scale and item analyses of Asian applications (Little et al., 2002; Rigotti et al., 2008). Subjective Well-being was measured using Keyes (2005) Subjective well-being instrument (adolescent version), which comprehensively assesses well-being in terms of emotional (3 items), psychological (4 items), and social (4 items) dimensions. All scales are shown in Table 1.

**TABLE 1:** Scale description.

Construct	Variables	Items
Student employability (SE)	General ability for work (GAW)	8 items
	Professional ability for work (PAW)	4 items
	Attitude at work (AW)	3 items
	Career planning and confidence (CPC)	3 items
Market orientation (MO)	Customer orientation	6 items
	Competitor orientation	4 items
	Inter-functional coordination	6 items
Teacher knowledge transfer (TKT)	Explicit knowledge	5 items
	Tacit knowledge	4 items
Absorptive capacity (AC)	Assessment	3 items
	Assimilation	3 items
	Application	3 items
Self-efficacy	Self-efficacy	6 items
Subjective well-being (SWB)	Emotional well-being (EWB)	3 items
	Psychological well-being (SWB)	4 items
	Social well-being (SoWB)	4 items

## Results

### Reliability and Validity

All scales used in this study were found to be reliable, with Cronbach’s α ranging from 0.65 to 0.92. Table 2 shows the reliability of each scale, and the factor loadings for each item therein. In order to gauge validity, this study employed confirmatory factor analysis (CFA) using AMOS 23.0 to verify the construct validity (both convergent and discriminant) of the scales. According to Hair et al. (2006) recommended validity criteria, CFA results show standardized factor loading of higher than 0.7; average variance extracted (AVE) ranges between 0.501 ∼ 0.806; and composite reliability (CR) ranges between 0.744 ∼ 0.918. All three criteria for convergent validity were met, and correlation coefficients were all less than the square root of the AVE within one dimension, suggesting that each dimension in this study had good discriminant validity.

**TABLE 2:** Measurement.

		1	2	3	4	5	6	7	8	9	10	11	12	13	14	15	16
(1) Customer		*0.80/0.71*	0.636**	0.562**	0.097*	0.128**	0.488**	0.398**	0.113**	0.050	0.080	0.099*	0.059	0.075	−0.010	−0.061	−0.015
(2) Competitor		0.760**	*0.83/0.76*	0.646**	0.053	0.094*	0.479**	0.466**	0.152**	0.059	0.073	0.066	0.059	0.044	0.016	−0.026	−0.003
(3) Coordication		0.712**	0.764**	*0.81/0.73*	0.021	0.034	0.533**	0.444**	0.090*	0.044	0.066	0.089*	0.149**	0.170**	0.130**	0.126**	0.177**
(4) Tacit		0.507**	0.412**	0.450**	*0.85/0.81*	0.651**	0.042	0.047	0.088*	0.040	0.009	−0.009	−0.008	0.025	−0.057	−0.068	−0.098*
(5) Explicit		0.498**	0.413**	0.464**	0.825**	*0.83/0.75*	0.046	0.048	0.143**	0.026	−0.056	−0.064	−0.055	0.020	−0.030	−0.084*	−0.109*
(6) Assessment		0.525**	0.508**	0.527**	0.441**	0.433**	*0.81/0.79*	0.479**	0.088*	0.045	0.107*	0.131**	0.111**	0.172**	0.061	0.035	0.121**
(7) Assimilation		0.527**	0.457**	0.460**	0.482**	0.458**	0.719**	*0.76/0.86*	0.395**	0.084*	0.056	0.072	0.071	0.120**	0.067	0.073	0.133**
(8) Application		0.538**	0.504**	0.467**	0.491**	0.449**	0.704**	0.668**	*0.83/0.78*	0.105*	0.054	0.047	0.034	0.053	−0.009	−0.061	−0.040
(9) GAW		0.518**	0.512**	0.521**	0.466**	0.494**	0.486**	0.462**	0.494**	*0.73/0.78*	0.143**	0.159**	0.118**	0.112**	0.042	0.097*	0.098*
(10) PAW		0.478**	0.496**	0.474**	0.451**	0.473**	0.445**	0.442**	0.461**	0.702**	*0.79/0.83*	0.616**	0.623**	0.125**	0.152**	0.113**	0.151**
(11) AW		0.559**	0.531**	0.536**	0.512**	0.544**	0.480**	0.471**	0.515**	0.649**	0.739**	*0.76/0.82*	0.699**	0.152**	0.224**	0.178**	0.233**
(12) CPC		0.538**	0.531**	0.527**	0.447**	0.475**	0.461**	0.440**	0.461**	0.651**	0.644**	0.734**	*0.82/0.90*	0.135**	0.194**	0.173**	0.210**
(13) Self-efficacy		0.498**	0.407**	0.438**	0.531**	0.535**	0.544**	0.574**	0.561**	0.506**	0.499**	0.548**	0.558**	*0.77/0.71*	0.326**	0.329**	0.360**
(14) EWB		0.420**	0.386**	0.434**	0.488**	0.456**	0.480**	0.417**	0.423**	0.400**	0.375**	0.475**	0.434**	0.555**	*0.87/0.88*	0.742**	0.646**
(15) PWB		0.487**	0.441**	0.474**	0.528**	0.531**	0.540**	0.510**	0.512**	0.514**	0.486**	0.522**	0.514**	0.687**	0.783**	0.80/0.82	0.728**
(16) SoWB		0.500**	0.471**	0.521**	0.448**	0.468**	0.488**	0.451**	0.456**	0.475**	0.442**	0.515**	0.500**	0.621**	0.681**	0.722**	0.84/0.84
Mean	Taiwan	3.54	3.46	3.40	3.89	3.85	3.54	3.71	3.64	3.53	3.62	3.60	3.54	3.75	3.63	3.71	3.51
	China	3.54	3.52	3.76	3.60	3.50	3.70	3.63	3.13	3.70	3.88	3.91	4.00	3.94	4.37	4.45	4.55
*SD*	Taiwan	0.65	0.71	0.70	0.66	0.66	0.66	0.64	0.68	0.64	0.70	0.70	0.72	0.62	0.72	0.68	0.77
	China	0.52	0.57	0.51	0.58	0.58	0.58	0.47	0.59	0.63	0.72	0.69	0.71	0.41	0.53	0.51	0.53
Cronbach’s α	Taiwan	0.91	0.89	0.92	0.91	0.92	0.85	0.81	0.87	0.90	0.87	0.79	0.86	0.90	0.90	0.88	0.90
	China	0.71	0.76	0.82	0.83	0.80	0.70	0.65	0.68	0.75	0.85	0.76	0.88	0.75	0.80	0.83	0.86
AVE	Taiwan	0.636	0.684	0.652	0.729	0.693	0.657	0.582	0.689	0.539	0.627	0.572	0.670	0.599	0.755	0.646	0.698
	China	0.501	0.584	0.527	0.661	0.556	0.621	0.739	0.611	0.604	0.695	0.677	0.806	0.501	0.781	0.669	0.701
CR	Taiwan	0.913	0.896	0.918	0.915	0.918	0.851	0.807	0.869	0.903	0.870	0.800	0.859	0.900	0.903	0.880	0.902
	China	0.806	0.848	0.870	0.886	0.862	0.831	0.850	0.823	0.744	0.901	0.863	0.926	0.830	0.884	0.890	0.907

Note: Italicized values mean square root of AVEs. *p < 0.1; **p < 0.01.

### Main Effect Analysis of the Structural Model

It is confirmed that the measurement pattern is stable. However, in order to avoid overgeneralizing the data-driven patterns and theories, the study follows the suggestion of Hair et al. (2006) to divide the sample data into two groups based on regions (657 Taiwanese and 565 mainland China students, respectively). Besides, multiple group testing is combined with bootstrapping to gradually control the pattern parameters of the groups. The nested models develop from the different limitations χ^2^ difference quantity to make significance analysis, in order to determine the reasonability of those parameters in controlling the two groups. The results are shown in Table 3.

**TABLE 3:** Multi-group testing.

Model	χ^2^	df	χ^2^/df	*p*	RMSEA	NFI	ECVI	0.9CI
(1) Unconstrained	780.23	308	2.533	0.000	0.036	0.948	0.835	(0.770 ∼ 0.906)
(2) Measurement weights	1054.68	320	3.296	0.000	0.044	0.93	1.043	(0.964 ∼ 0.1.128)
(3) Structural weights	1147.62	328	3.499	0.000	0.046	0.924	1.107	(1.024 ∼ 1.196)
(4) Structural covariances	1483.71	340	4.364	0.000	0.053	0.901	1.366	(1.270 ∼ 1.469)
(5) Structural residuals	1565.72	342	4.578	0.000	0.055	0.896	1.431	(1.332 ∼ 1.536)
(6) Measurement residuals	2195.68	362	6.065	0.000	0.065	0.854	1.922	(1.802 ∼ 2.047)
2–1	274.45	12		0.000		0.018		
3–1	367.38	20		0.000		0.024		
4–1	703.48	32		0.000		0.047		
5–1	785.49	34		0.000		0.052		
6–1	1415.44	54		0.000		0.094		

The analysis results show that the value of each pattern mode of χ^2^/*df* ranges from 2.533 to 6.065, the RMSEA ranges between 0.036 and 0.065 and the ECVI is within 90% of the confidence interval. It can be learned from Table 3 that the χ^2^ values of the weighted measurement model, weighted structure model, covariance structure model and residual structure model reach significant levels, which shows that the models have good between-groups invariance. In addition, the NFI added value of each model was less than 0.05, which is consistent with the standard recommended by Little (1997). Therefore, the framework and conclusion of this research will present a good generalized validity.

**FIGURE 2:**
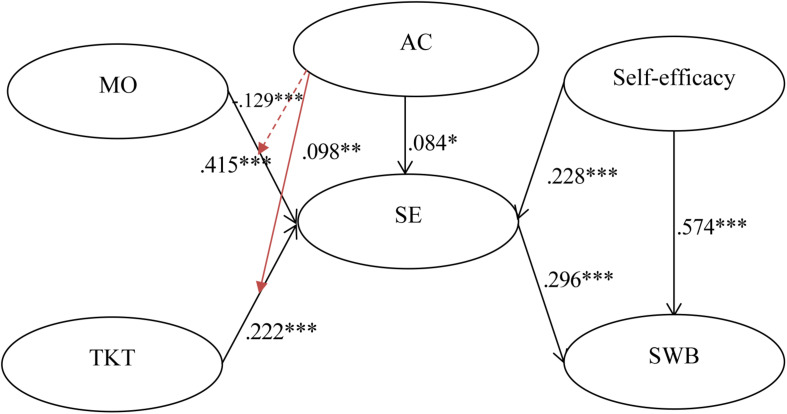
Structural model on Taiwanese students. ^∗^*p* < 0.05, ^∗∗^*p* < 0.01, ^∗∗∗^*p* < 0.001.

**FIGURE 3:**
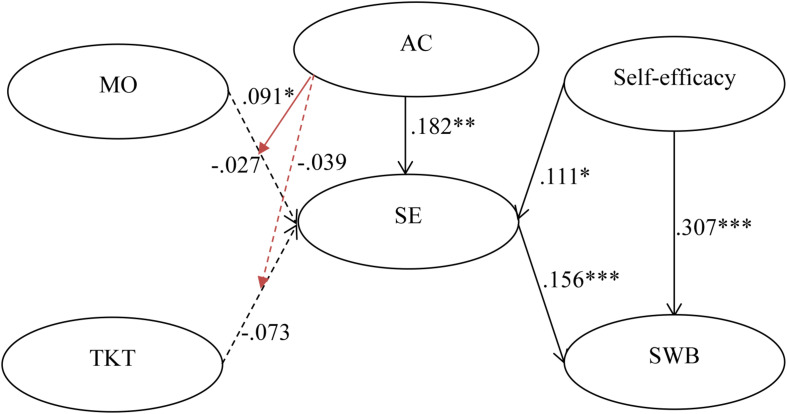
Structural model on Taiwanese students. ^∗^*p* < 0.05, ^∗∗^*p* < 0.01, ^∗∗∗^*p* < 0.001.

The multi-group analysis method recommended by Chin (2004) was utilized to examine the hypothesis on the moderating effects of each country in the research model. The path coefficients and *t*-values of the hypothesized relationships were calculated to evaluate the significance of the relationships in each subgroup. The standardized structural weights for Taiwan and mainland China are shown in Table 4, Figures 2, 3. These standardized structural weights were estimated with the item-factor loadings held equal across countries. Thus, they are the best estimates of the true structural weights. Table 4 shows that seven hypotheses were supported for the Taiwanese subgroup, whereas for the Chinese subgroup only five hypotheses were supported. As shown above, H4 and H5 were partially supported, while H1, H2, H3, and H6 were fully supported. The results in Table 3 also show the addition of the interaction terms to the main effects model of the Taiwan and mainland China samples. In H7, we argue that interaction term of MO and AC has a positive effect on SE. Although interaction terms of both samples were significant, but coefficients of the Taiwanese sample were negative (−0.129 and 0.091). Consequently, H7 is partially supported. With regard to H8, Table 4 shows that the interaction term between TKT and AC moderates positively to SE (β = 0.098, *p* < 0.05) in the Taiwanese sample. Therefore, our findings partially support H8.

**TABLE 4:** Comparison analysis between Taiwan and Mainland China.

	Taiwan		Mainland China		
Path	Standardized path coefficient	Standard errors for structural path	Standardized path coefficient	Standard errors for structural path	*t* Statistic (two-tailed)
MO → SE	0.415***	0.047	−0.027	0.191	52.60***
KT → SE	0.222***	0.042	−0.073	0.040	119.06***
AC → SE	0.084^†^	0.049	0.182**	0.085	−23.38***
Self-efficacy → SE	0.228***	0.051	0.111**	0.074	30.47***
Self-efficacy → SWB	0.574***	0.050	0.307***	0.056	83.25***
SE → SWB	0.296***	0.043	0.156***	0.034	59.78***
AC*MO → SE	−0.129***	0.014	0.091*	0.017	−233.84***
AC*TKT → SE	0.098**	0.016	−0.039	0.020	125.21***

^†^p < 0.1, *p < 0.05, **p < 0.01, ***p < 0.001.

The statistical analysis shows that there were differences between the Taiwanese and Chinese subgroups. The objective was then to determine whether the differences were significant or not. First, the data were tested using the Kolmogorov–Smirnov test of normality, and the results indicated that it was not distributed normally. Therefore, the Smith–Satterthwaite *t*-test, which is utilized when the data violate normal distribution or exhibit unequal variances, was selected (Chin, 2004). Thereafter, the results of the *t*-tests for each subgroup are detailed in Table 3. There were significant differences in the all eight path coefficients between the two subgroups. Except for the path coefficient of AC → SE and AC^∗^MO → SE between the two subgroups were negatively significant differences, the remaining six path coefficients produced positively significant differences. The results of hypothesis testing are discussed further in the following sections for their possible implications for teaching.

## Conclusion

### Research Findings and Discussion

This study takes university students as research samples to test the correlations among MO, TKT, AC, SE, self-efficacy, and SWB by using the competition enhancement model, thus to bridge the theoretical gap of applying western theory in the eastern situation, and to increase the generalization of learning cognition theory. This study also finds the crux of the real-world phenomena based on the suggestions of scholars and verifies the differences between the variables of Taiwanese and mainland Chinese samples within the research structure (Lu et al., 2003; Lu, 2009). Based on the research results, this study proposes the following research findings.

First, the study found that regarding the correlation between the research frameworks in the mainland Chinese sample, only the employability has a positive impact on self-efficacy and happiness. It means that even in the context of the East to verify the theoretical framework proposed by the Institute, different countries may also produce different outcomes, namely the potential interference effects of differences in social systems. The result have brought deeper enlightenment to the research in the field of education. When we use Western theories to verify their applicability to students in Eastern contexts, different social systems will also produce different results. Therefore, in addition to cultural differences, comparisons between countries are also necessary (Chang et al., 2011).

Second, under the Taiwan sample, the correlations among the variables in the research framework are positive and statistically significant, meaning that Taiwanese students’ efforts in collecting market information and TKT are helpful to improve SE. When students are highly employable, they are more confident in psychological cognition to face all challenges, thereby enhancing their self-efficacy and SWB. This result is consistent with the research results of Cuyper et al. (2008) and Dacre and Qualter (2013). However, for the mainland Chinese samples, MO and AC have no statistically significant impact on SE; in fact, TKT even has a significantly negative impact, which is inconsistent with this study’s inference. The possible reason is students in mainland China have a strong spontaneous learning attitude. They believe that the acquisition of skills and knowledge come from the process of self-study. Therefore, even though the ability to collect and absorb employment market information has become a tool for students to learn by themselves, the effect is not significant.

Thirdly, from the difference analysis of path coefficients, it is known that except for absorption capacity, the difference of the SE path is not significant; the other paths are statistically significant, representing the correlation between all paths, and the degree of Taiwanese students’ feelings will be larger than mainland Chinese students. Taiwanese students are aware of a high degree of personal obligation in the face of TKT and employment market information collection. Therefore, it is easy to detect the role that they should play as having a positive impact on the participation of various learning activities.

Fourth, this study infers that the individual’s AC will positively strengthen the influence of MO and TKT on SE. The study found that for the student aspect of Taiwan, the interaction effect between TKT and AC has a positive moderating effect on employability; it represents the assertion that AC can help to enhance the influence of TKT activities on SE. However, it is interesting to note that the interaction between MO and AC has a significant negative impact on Taiwanese SE, while conversely having a positive impact on students in mainland China. It implies that if students have higher AC, it will weaken the positive effect of MO on SE. This differs from the claims of Webster and Hommond (2008) and Webster et al. (2014). Furthermore, although the employability of students in Taiwan can benefit from MO and TKT, students still have to apply knowledge to their internal capacity through the teacher’s introduction and explanation of knowledge. Even if students can obtain a large amount of external information, if there is no appropriate knowledge base and ability foundation, students will face learning obstacles which will reduce the positive impact on SE.

### Educational Implications

This study explores the enhancement of employability development between Taiwanese and mainland Chinese students from the perspective of capacity enhancement. It is found that SE is the key to students’ self-efficacy and SWB, and the shaping of SE has considerable influence on the establishment of external information and TKT. In the process of education, schools and their teachers should identify the students’ thinking patterns and behavioral rules, and integrate the employment market perception into the students’ learning connotation, in order to successfully shape the market-oriented culture and strengthen their keen awareness of external information to meet the high expectations and employment conditions of future employers. On the other hand, it is known from the research findings that although Taiwanese students are better than mainland students in shaping their employability, the active self-learning attitude still needs to be strengthened. Therefore, it is more important for teachers to guide students to develop their employability through TKT activities.

In addition, the individual’s AC has no significant difference in the path of employment between the Taiwan sample and the mainland Chinese sample, indicating that AC plays an important role in shaping and developing students’ abilities regardless of the situation. In order to realize the benefits of AC, students must have a solid foundation of knowledge in order to clearly translate new knowledge and external information into their own understanding of knowledge and subsequently apply their employment skills (Nor et al., 2012). Therefore, this study suggests that schools should assist students in establishing their own knowledge bases and effective knowledge-processing processes with knowledge learning platforms or electronic databases in order to simplify complex implicit and explicit knowledge and improve their employment skills.

The study found that Taiwanese college students are superior to mainland students in terms of the impact of the acquisition of their employability or the improvement of their self-efficacy on SWB. In other words, although students need more employment skills to face the uncertainty of the future employment workplace, students can gain more self-recognition in terms of self-growth, independence, etc., or can perceive that they contribute more socially. They can resonate with society and be accepted by society, and the feelings of self-emotion are happy and satisfied, making challenge of attaining knowledge have a positive influence on students. Therefore, this study suggests that Taiwanese universities should provide students with opportunities to develop superior employability in teaching activities and curriculum design, and strengthen self-belief, practical ability, professional ethics learning and application opportunities, so that students have higher psychological, social and emotional satisfaction (Keyes, 2005) growing from learning in an appealing environment.

In short, Taiwanese and mainland students have their own merits at differing levels under this research structure. For example, Taiwanese students have diverse ideas and practical application experience, but lack clear learning goals and positive learning attitudes. Mainland students have strong motivation for learning and effective methods of knowledge exploration, but the abilities of problem-solving and creativity are slightly deficient. Therefore, if we can strengthen cross-strait teacher-student exchanges, through extensive short-term training, observation and academic competitions, and in-depth discussions on teacher curriculum and student learning outcomes, it will help each other in mutual learning and growth, thus improving the learning efficiency and employment competitiveness of cross-strait college students.

### Research Limitations and Future Research Directions

The MO, TKT, SE, self-efficacy, and SWB scale of this research structure are based on the same set of samples for the reliability and validity test of the question scale, although Hair et al. (2006) agree that a same set of samples can be used for EFA and CFA to develop a measure of constructs. If, however, we can use another new set of samples for cross-validation, the reliability and validity of the study scale should be more valid. In addition, it is recommended that future researchers can expand the number of samples or compare and analyze them in different regions in order to better understand the differences. Moreover, higher education is not a single market. In addition to students, it also includes stakeholders such as teachers, staff, and employers. However, this study only explores students’ self-awareness of employability. It does not mean that their knowledge and skills are in line with the expectations of society and employers. Therefore, it is suggested that the measurement of SE in the future can be compared with each other, and the influence of different groups of cross-level factors will be explored. Additionally, it will be helpful to understand the cognitive differences between the students and the employment market.

## Data Availability Statement

The raw data supporting the conclusions of this article will be made available by the authors, without undue reservation, to any qualified researcher.

## Ethics Statement

The studies involving human participants were reviewed and approved by Institutional Review Board, University of Taipei. The patients/participants provided their written informed consent to participate in this study.

## Author Contributions

YX and MP contributed to the ideas of educational research, collection of data, and empirical analysis. MP, YS, S-HW, and W-LC contributed to the data analysis, design of research methods, and tables. MP and C-CL participated in developing a research design, writing, and interpreting the analysis. All authors contributed to the literature review and conclusions.

## Conflict of Interest

The authors declare that the research was conducted in the absence of any commercial or financial relationships that could be construed as a potential conflict of interest.
